# Machine learning and metabolic modeling-based identification of hypoxia-driven metabolic signatures in pediatric cancers

**DOI:** 10.3389/fphar.2026.1810370

**Published:** 2026-04-30

**Authors:** Subasree Sridhar, G. K. Suraishkumar

**Affiliations:** Department of Biotechnology, Bhupat and Jyoti Mehta School of Biosciences Building-1, Indian Institute of Technology Madras, Chennai, India

**Keywords:** genome-scale metabolic models, hypoxia, pediatric cancers, reactive oxygen species, reactive sulfur species, machine learning

## Abstract

Metabolic reprogramming in pediatric cancers under hypoxia has been much less studied compared to adult-onset cancers; such studies are essential to identify relevant therapeutic targets at different hypoxic levels. Genome-scale metabolic modeling studies of cancer metabolism are used to elucidate reprogrammed metabolic pathways, analyze heterogeneity between cancer types and subtypes, identify synthetic lethality, perform *in silico* drug targeting, etc. We have used reactive-species-integrated genome-scale metabolic models of ten different pediatric cancer cell lines to understand metabolic rewiring under hypoxia. These pediatric cancer models exhibited a consistent metabolic signature under the given constraints, under each oxygen condition. We applied constraint-based modeling to simulate normoxic, hypoxic, and extreme hypoxic gradients and used machine learning to analyze the metabolic pathways that predominate across these gradients in pediatric cancers. Our machine learning classifiers showed improved accuracy in classifying the different gradients and generated metabolic features of importance. We performed a reaction-level flux analysis on the predicted discriminative metabolic features of importance. We observed that most metabolic pathways are not linearly rewired when hypoxic levels increase. Hypoxia-specific metabolic rewiring is enriched with reactive oxygen species, and extreme hypoxic cancer states exhibit a positive contribution from mitochondrial reactive sulfur species. Further, we propose that reactive sulfur species and reactive oxygen species detoxifying reactions contribute to redox balance in extreme hypoxia and hypoxia, respectively.

## 1 Introduction

Pediatric cancers develop mainly from developmental tissues, while adult cancers originate from epithelial tissues due to accumulated mutations over the years (Chen et al., 2024); pediatric cancers harbor fewer mutations than adult-onset cancers (Gröbner et al., 2018). This primary difference necessitates an in-depth investigation of metabolic reprogramming in pediatric cancers. Most cancer studies with constraint-based modeling have focused on adult-onset cancers. We have chosen 10 common pediatric cancers for our study. Tumor cells employ different adaptive mechanisms at different hypoxic levels (Hompland et al., 2021). Only two genome-scale metabolic modeling studies have elucidated hypoxic-mediated reprogramming in adult-onset cancers using omics data (Damiani et al., 2019; Omer et al., 2025). The first study relied on bulk and single-cell transcriptomic data to infer fluxes and used hypoxia signature scores to relate oxygen and glucose uptake rates and lactate secretion rates in cancers. The second study utilized proteomics data under normoxic and hypoxic conditions and used gene-protein-reaction rules in genome-scale metabolic models (GEMs) to constrain the reactions according to hypoxic conditions in a colorectal cancer cell line GEM. However, Eyassu and Angione (2017) applied constraints on the oxygen uptake reaction to simulate normoxic and hypoxic conditions in a generic cancer metabolic model and used an unsupervised machine learning algorithm to analyze hypoxia driving forces. Others, such as Wilson et al. (2025); Çakιr et al. (2007), have used constraints on oxygen uptake and hypoxia-mediated biochemical constraints to simulate hypoxia in astrocyte GEMs, respectively. In this study, we simulated normoxic, hypoxic, and extreme hypoxic states in pediatric cancer GEMs by constraining oxygen-associated reactions in our pediatric GEMs without using any hypoxic-specific expression data.

The explosion of omics data has expedited the integration of Machine Learning (ML) into constraint-based modeling studies (Zampieri et al., 2019; Antonakoudis et al., 2020; Lewis and Kemp, 2021). The scope, framework, and basic guidelines for the analysis of metabolic modeling integrated with ML were explained by Vijayakumar et al. (2022). Machine learning techniques have enabled the integration of omics and image data into constraint-based metabolic models to infer flux outputs and predict metabolic shifts in cancers compared to normal tissues, quantify quiscense depth for phenotype prediction, and identify therapeutic targets in cancers, respectively (Yang et al., 2023; Eames and Chandrasekaran, 2024; Tavakoli et al., 2025). Zehetner et al. (2024) formulated logistic principal component analysis to differentiate GEMs based on model compositions. Woo et al. (2024) used ML algorithms on genome-wide gene-deletion data and flux data to predict essential reactions under different carbon sources. Czajka et al. (2021) accurately predicted chemical titers in high-yielding strains of *Yarrowia lipolytica* by integrating ML with metabolic modeling and knowledge mining. Chung and Chandrasekaran (2022) used ML on heterogeneous pathogen flux datasets to predict the drug interaction type. Razmpour et al. (2026) generated synthetic healthy samples using a Generative Adversarial Network for comparison against pancreatic tumor transcriptomic datasets, and used them for constructing GEMs. These were further used to elucidate the mechanism in pancreatic cancer GEMs. In this work, we constrained oxygen uptake and consumption rates of pediatric cancer GEMs with experimental data to simulate normoxic, hypoxic, and extreme hypoxic conditions. We used different, supervised, classification-based ML algorithms to analyze pediatric cancer GEMs using flux data. We further investigated metabolic pathways that play key roles in the classification of three oxygenated states in pediatric cancers.

## 2 Methods

### 2.1 GEMs of pediatric cancers

The details of GEMs belonging to 10 pediatric cancers (Kumar et al., 2026) shown in Supplementary Section 1 were reconstructed using transcriptomics data taken from Cancer Cell Line Encyclopedia (CCLE) (version: DepMap Public 23Q4) (Arafeh et al., 2025; Dharia et al., 2021; Sun et al., 2023). Kumar, S. et al. reconstructed the Recon 3D-derived cancer cell line GEMs using SPECTRA, a framework for reconstructing metabolic models from multi-omics data, and *Localgini* was used for thresholding (Brunk et al., 2018; Kumar and Bhatt, 2025). This version of the CCLE data includes around 180 cell lines for 23 pediatric cancers, of which only 10 cancers have a comparable distribution of cell lines. There are 147 cancer cell lines and their derived metabolic models used in our study (Supplementary Tables S1,S2). Complete details of the 147 cell lines are provided in Supplementary Table S1. We used COBRA Toolbox (MATLAB®) for modification of GEMs (Heirendt et al., 2019). Basal Medium Eagle (BME) media constraints were applied to these GEMs. The details about BME constraints are shared in Supplementary Section 2. The BME constrained cancer GEMs were then integrated with the reactive species module that we had developed earlier (Sridhar et al., 2023), to account for all reactive species reactions specific to cancer metabolism. Artificial reactions in the module, such as exchange, demand, and sink reactions involving the reactive species, were removed to ensure that the reactive species are not generated from nothing. Thermodynamically infeasible cycles and duplicate reactions were also removed from the integrated models. We have added compartment-wise demand reactions and a total cellular demand reaction for about 12 reactive species, including hydrogen peroxide, superoxide, hydrogen sulfide, and hypochlorous acid. The details about the demand reactions are shared in Supplementary Section 4. These media-constrained and reactive species integrated metabolic models were checked for the activity of biomass growth, energy metabolism, biosynthesis of certain metabolites like biomass precursors, substrate utilization, and internal conversion processes, etc., that represent basic human metabolic tasks (Heirendt et al., 2019). The presence of core cancer hallmark reactions involved in the Warburg and proliferation was also confirmed (Dai et al., 2019). We found that most of our models have activity in around 77.83% of predefined tasks. Most of our models have 75.56% of the hallmark reactions in them. The codes used for the modification and analysis of GEMs discussed in this section are available in the provided Zenodo repository.

### 2.2 Simulation of normoxia, hypoxia, and extreme hypoxia states

The 147 cancer GEMs were constrained with normoxic to extreme hypoxic states with 10 fractions under 3 broad labels (normoxia, hypoxia, and extreme hypoxia). For the normoxic state, the maximum allowable uptake bound for oxygen was set at 1.19 mmol/gDW/h (Eyassu and Angione, 2017). Optimal oxygen uptake estimated using flux-balance analysis in the normoxic state was 10-fold less than 1.19 mmol/gDW/h (Orth et al., 2010). Oxygen is not a growth-limiting factor in large GEMs such as cancer cell lines, so an additional constraint on cytochrome oxidase reaction (complex IV enzyme) was also added. The bounds of cytochrome oxidase were constrained by experimentally measured basal oxygen consumption rates (OCR) from cancer cells (Takahashi and Yamaoka, 2017). We have generated 10 fractions of oxygen limitation constraints on oxygen uptake and consumption rate reactions to denote three states: normoxia, hypoxia, and extreme hypoxia. Normoxia encompasses two oxygen gradients, 100% and 50%. Hypoxia and extreme hypoxia consist of 4 gradients each; hypoxia encodes for 20% 10% 5% and 2% whereas extreme hypoxia encodes 1% 0.5% 0.2% and 0.1% (Hompland et al., 2021). This approach allows the models to utilize gradual metabolic adaptations that occur as oxygen availability decreases. Based on the classification of oxygen fractions, the number of normoxia samples is 294, and the number of hypoxia and extreme hypoxia samples is 588 each.

### 2.3 Flux analysis of GEMs

Parsimonious flux balance analysis (pFBA) of the 147 GEMs across the 10 oxygen gradients was performed using COBRApy in PYTHON® (Ebrahim et al., 2013). pFBA maximizes the flux through the biomass reaction by minimizing the sum of the fluxes through the remaining metabolic reactions under applied constraints (Lewis et al., 2010). Biomass reaction was chosen as the objective function at all gradients. We selected reactions with non-zero pFBA flux values and values above the model tolerance for our analysis. All of these reactions are essential for biomass optimization. We decided to use pFBA for our analysis because it provides the exact metabolic dependencies under hypoxia, which can be targeted. It is also known that pediatric cancers depend on fewer metabolic pathways for their sustenance (Issaq and Heske, 2020).

#### 2.3.1 Pediatric cancers consistency analysis

We assessed whether all 10 pediatric cancers exhibit metabolic consistency under each oxygen state. In the Recon 3D-derived metabolic models, reactions are grouped into metabolic pathways. We calculated the ratio of pathway fluxes to the total flux for each GEM under each oxygen condition for all available pathways. We selected median pathway flux fractions per oxygen state for cell line GEMs in each cancer category for the 10 pediatric cancers. We also measured global metabolic signatures by averaging all the pathway flux fractions separately for each oxygen condition in 10 cancer categories. A Spearman correlation analysis was performed between each cancer type’s median normalized flux fraction profile and the global profile across the 3 oxygen conditions. Spearman correlations were computed using scipy.stats.spearmanr (Pedregosa et al., 2011). Results are visualized as a ranked horizontal bar chart with cancer categories color-coded by oxygen fractions, given in Supplementary Figure S1. The codes used for the analysis discussed in this section are available in the provided Zenodo repository.

### 2.4 Supervised machine learning analysis

**FIGURE 1:**
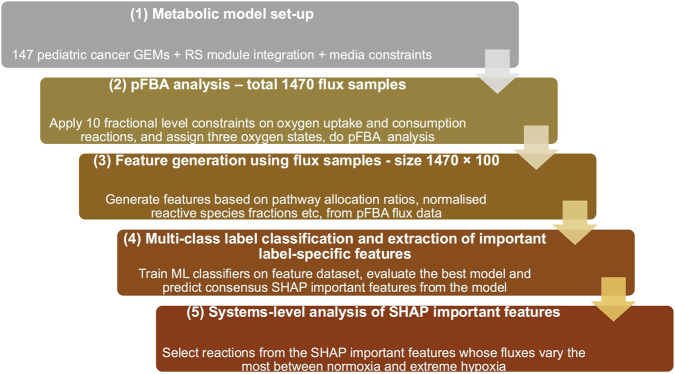
Detailed workflow of integrating genome-scale metabolic modeling with machine learning. The pipeline consists of 5 major steps. (1) Model modification for 147 pediatric cancer cell lines GEMs, (2) Generation of parsimonious flux samples for normoxia, hypoxia, and extreme hypoxia under suitable constraints, (3) Generation of features based on pathway allocation and ratios from the flux samples, (4) Training and evaluation of machine learning models and extraction of features of importance useful in classification from the best model and finally (5) Systems-level analysis of reactions belonging to the SHAP important features whose fluxes varied the most between normoxia and extreme hypoxia.

We used supervised ML analysis of pFBA values from 147 GEMs of pediatric cancer cell lines to uncover biological pathways that distinguish metabolic pathways across 10 oxygen fractions. The total number of flux samples across 147 models in 10 oxygen fractions is 1470. We used scikit-learn v1.8.0 library for all the ML analysis, and we started with 6 well-known classification-based ML models suitable for our objective (Pedregosa et al., 2011). We derived features based on four types: (i) fractional contribution of each pathway flux to total metabolic flux activity, (ii) proportion of oncometabolite exchange flux to total exchange fluxes, (iii) ratio of reactive species demand flux to total metabolic flux, and (iv) ratio of a few pathway fluxes to capture metabolic trade-offs. The total number of features is 100. The details of the feature matrix (dataset) used in the analysis are given in Supplementary Section 4. The detailed workflow adopted to differentiate the three oxygen gradients based on metabolic features derived from pFBA values is shown in Figure 1. The codes used for the ML analysis are available in the provided Zenodo repository. Given below are the detailed steps of the ML workflow

**FIGURE 2:**
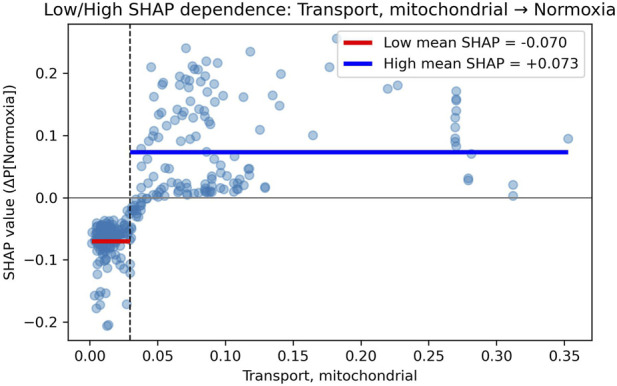
SHAP dependence plot for a pathway in normoxia prediction. The pathway shown here is Transport, mitochondrial, which includes transport reactions for metabolites between mitochondria and cytosol. Each point represents a sample (cell line under a normoxia constraint) of the test set, showing the relationship between feature value (x-axis; aggregated pathway flux ratio) and its corresponding SHAP value (y-axis), which quantifies the contribution of that feature to LightGBM prediction of normoxia. Positive SHAP values indicate increased probability of normoxia prediction, whereas negative values indicate reduced probability. These plots reveal how variation in pathway-level fluxes influences classification across oxygen states, highlighting nonlinear and threshold-dependent effects in metabolic responses.

**FIGURE 3:**
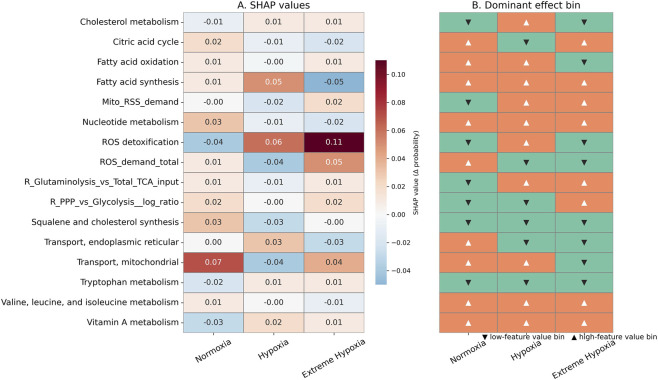
Dominant-bin SHAP effects of consensus important features on oxygen state classification. Heatmap showing the mean SHAP value ( Δ probability) of the dominant feature bin for each of the 16 consensus SHAP-ranked metabolic features across the three oxygen states - normoxia, hypoxia, and extreme hypoxia. For each feature, SHAP dependence plots were used to stratify samples into low-value and high-value bins based on the median feature value, and the dominant bin was defined as the bin with the higher absolute mean SHAP value. Positive SHAP values indicate that the dominant bin increases the predicted probability of that oxygen state, while negative values indicate a decrease. Features whose absolute values are equal to or greater than 0.01 are further discussed. **(A)** SHAP value. **(B)** Dominant effect bin.

1. We split 80% of the data (1470 samples) into the training set and the remaining 20% into the test. In the 80% of the total training set, we split 75% as training and the remaining 25% as a validation set, respectively. All data splits were performed using stratified shuffle splitting, where the proportion of normoxia, hypoxia, and extreme hypoxia was kept similar to their proportion of the entire dataset.
2. We chose some well-known ML algorithms for classification (Logistic Regression, Support Vector Machines, Random Forest (RF), GradientBoosting (GB), HistGradientBoosting (HGB)), and trained Light Gradient Boosting Machine (LightGBM), and trained them on the training data. We selected the top 3 models based on their performance on the validation set. We used a performance metric called the macro-averaged F1 score, which computes the mean of the unweighted F1 scores for each label and is useful in our case, where we have a moderately imbalanced dataset (Sokolova and Lapalme, 2009).
3. We improved the performance of the top three models by tuning hyperparameters to achieve better classification results using grid search with stratified 5-fold cross-validation, evaluated using macro-averaged F1 scores on the full training set (training plus validation).
4. The three models with tuned hyperparameters were finally tested on the test data set, and the performances of the models were evaluated using averaged macro-F1 scores. macro-AUC score and AUC-ROC (Area Under the Receiver Operating Characteristic Curve). Macro-AUC score and AUC-ROC measure a model’s ability to distinguish between the different states across different thresholds of classification, in a one versus rest approach (Bradley, 1997; Hand and Till, 2001). AUC values range from 0 to 1, where a score near 1 indicates an excellent classifier.
5. Importance of individual features toward the prediction of each class was determined by calculating the SHapley Additive exPlanations (SHAP) values (Lundberg and Lee, 2017). It tells us which features, by how much, push or pull the model towards predicting each class. In our case, we used SHAP values to distinguish between normoxia, hypoxia, and extreme hypoxia on our test data. The top-ranked global features of importance were estimated by ranking based on their mean absolute SHAP values predicted by the top 3 ML classifiers. Then, the top consensus features were selected from this combined list. We plotted SHAP dependence plots between SHAP values vs. original feature values of these consensus features across each label (an example of a dependence plot for a topmost feature vs. its SHAP values in normoxia is shown in Figure 2. The two entities exhibited a non-linear relationship; to better interpret this, we divided the features into low and high regimes based on their median. We calculated mean SHAP values across the two regimes and chose the regime with the higher absolute mean value (the dominant bin) to interpret the effects of the features on label classification. In Figure 2, the dominant bin is the higher regime (with a positive value) because its absolute mean SHAP (0.073) value is higher than that of the low regime (−0.07). We then plotted a heatmap for the consensus features across three oxygenated states to depict the effects of features in the dominant bin on class prediction, shown in Figures 3A,B. For SHAP relationships explained in the results section, the signs of the mean SHAP values in the low and high regimes are opposite, and the magnitudes differ, too. This is important because when we say that a feature with low value (low regime) has a negative mean SHAP, it means that low values of that feature decrease the probability of that class being predicted. This is consistent with saying higher values of the same feature weakly increase the probability of that class. For our results, we discuss only those features with absolute mean SHAP values greater than 0.01.

#### 2.4.1 Systems-level flux analysis

**FIGURE 4 F4:**
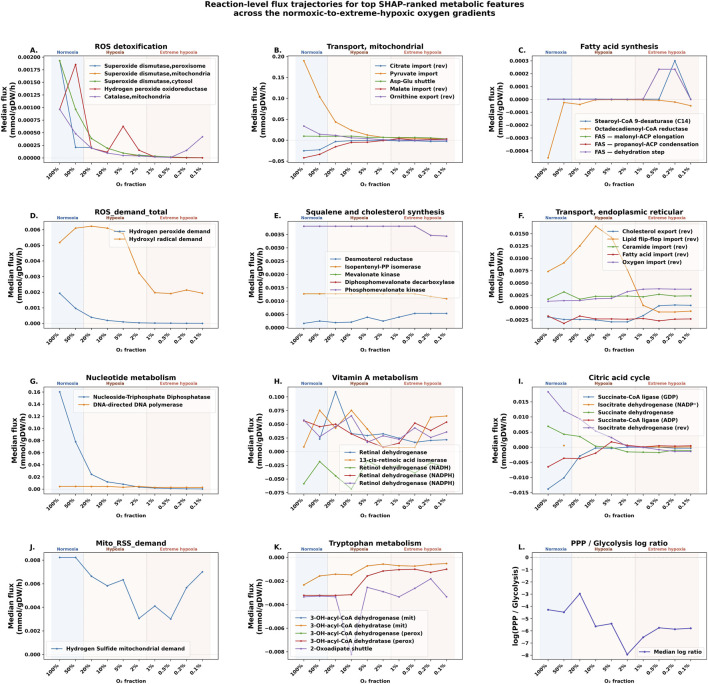
Reaction-level flux trajectories from consensus SHAP-ranked metabolic features across the normoxic-to-extreme-hypoxic oxygen gradient in pediatric cancer genome-scale metabolic models. Each panel shows the median signed flux (mmol/gDW/h) of selected reactions within each top SHAP feature across 10 oxygen fractions spanning normoxia (100%,50%) , hypoxia (20%,10%,5%,2%) , and extreme hypoxia (1%,0.5%,0.2%,0.1%) . Vertical dashed lines denote class boundaries between normoxia, hypoxia, and extreme hypoxia. Features are ordered by maximum absolute dominant-bin SHAP value from **(A–L)**. For Transport, mitochondrial and Transport, endoplasmic reticular, reactions were selected based on biological relevance to cancer metabolism. For all other pathways, reactions were selected by the coefficient of variation of median signed flux across the oxygen gradients. Positive flux values indicate forward reaction; negative values indicate reverse (depends on the directionality of annotated reactions in GEMs). Trajectories represent median flux values of reactions across 10 pediatric cancer categories in each of the oxygen fractions. Reactions shown in legends, but not in trajectories, are active only in one of the 10 fractions, denoted as a dot.

We performed a systems-level flux analysis using the original pFBA fluxes of the 147 genome-scale metabolic models across all 10 oxygen fractions to understand the biological basis of the SHAP analysis. We plotted reaction-level trajectory plots to characterize flux changes across the 3 states distributed over 10 oxygen fractions shown in Figure 4. We plotted only the top consensus SHAP-ranked metabolic features that are greater than or equal to 0.02 in at least one oxygen state. We plotted those reactions whose fluxes varied strongly between normoxia and extreme hypoxia. To this end, we used a metric called the coefficient of variation for the median signed fluxes across the 10 oxygen fractions. Median signed pFBA flux values across all cell lines were plotted for the 10 oxygen fractions. The codes used for the systems-level analysis are available in the provided Zenodo repository.

## 3 Results

We used pFBA in our comparative analysis of 147 reactive-species-integrated pediatric cancer cell line GEMs, as it gives the minimum essential reactions needed to sustain growth under the given constraints. We concluded from the Spearman correlation analysis that the metabolic signatures identified were broadly conserved across all ten pediatric cancer categories. Each of the 10 cancers exhibited high metabolic consistency in normoxia, where the correlation coefficient ρ ranged between 0.79−0.91 , hypoxia ranged between 0.85−0.91 , and extreme hypoxia ranged between 0.75−0.9 , (Supplementary Figure S1). More details about the results are provided in Supplementary Section 3. We used ML techniques to compare 1470 pFBA flux samples from 10 pediatric cancers across normoxia, hypoxia, and extreme hypoxia.

### 3.1 Details of ML model performance in classification

We found that the three ensemble learning algorithms, LightGBM, HGB, and RF algorithms had overall higher macro-averaged F1 scores and AUC scores than the other algorithms. HGB and LightGBM are boosting-based, and RF is a bagging-based ensemble algorithm. Among these three, we have chosen the LightGBM algorithm for further SHAP analysis based on their evaluation metrics on test data over the other two. The plots of evaluation metrics of LightGBM are given in Supplementary Figure S2. The plots of evaluation metrics of RF and HGB are given in Supplementary Section 5, Supplementary Figure S3 for HGB and Supplementary Figure S4 for RF. The LightGBM-derived SHAP value of each feature in our test data explains how much the class probability changes for that feature. Probability-based SHAP values are more representative of biology, which is useful in our case.

### 3.2 Driving forces at different oxygenated states of pediatric cancers

We will now focus on the most important features that support the prediction of a specific class and distinguish the 3 oxygenated states in pediatric cancers, as revealed by their SHAP values. As explained in the methods section, we took the top features, as predicted by the top three models, for classifying three labels.

#### 3.2.1 Key features that contribute to the classification of each oxygen state

Features that increase the probability of predicting a specific class are the state-specific metabolic dependencies of that oxygenated state, shown in Figures 3A,B.

1. Normoxia: We observe that higher values of pathway allocation of citric acid cycle (TCA cycle), fatty acid oxidation, fatty acid synthesis, nucleotide metabolism, and valine, leucine, and isoleucine (branched-chain amino acid metabolism) increase the probability of normoxia prediction by the model. Similarly, features like cholesterol metabolism, reactive oxygen species (ROS) detoxification, and tryptophan metabolism are compatible with normoxia classification. Lastly, total ROS demand and mitochondrial transport, which denotes transport between mitochondria and the cytosol, are positively associated with the classification of the normoxic state.
2. Hypoxia: We observe that higher cholesterol metabolism and fatty acid synthesis from our samples push the prediction to hypoxia. Similarly high value of ROS detoxification from our samples increases the probability of hypoxia. The same is true for Vitamin A metabolism. A hypoxic state shows a positive association with cholesterol synthesis, total ROS demand, and the citric acid cycle; low values of these features decrease the probability of hypoxia prediction, and *vice versa*.
3. Extreme hypoxia: We observed that mitochondrial reactive sulfur species (RSS) demand is a key signature of extreme hypoxic conditions. We observe that two defined features, which are the log ratios of two pathways: (a) pentose phosphate pathway (PPP)/glycolysis, and (b) glutaminolysis/total TCA input, are positively associated with extreme hypoxia prediction. PPP/glycolysis measures the fraction of glucose devoted to NAD(P)H generation compared to ATP synthesis. Glutaminolysis/total TCA input quantifies the glutamine-dependent anaplerosis that replenishes the TCA cycle. Enhanced PPP and glutaminolysis in the samples push the ML classifier to predict an extreme hypoxic state. Extreme hypoxia classification is positively associated with pathways like vitamin A and endoplasmic reticulum (ER) - cytosol transport.

Let us now focus on features that have antagonistic effects on classification across three labels, as seen in Figures 3A,B.

- High levels of vitamin A in samples decrease the probability of predicting normoxia. Low values of features like PPP/glycolysis, and cholesterol synthesis increase the probability of normoxia prediction
- High values of features like mitochondrial RSS demand, nucleotide metabolism, glutaminolysis/total TCA cycle input, and mitochondria - cytosol decrease the probability of hypoxia prediction. Low values of endoplasmic reticulum - cytosol transport and tryptophan metabolism increase the probability of hypoxia prediction.
- Low values of cholesterol metabolism, fatty acid oxidation, ROS detoxification, total ROS demand, and tryptophan metabolism increase the probability of extreme hypoxia prediction. High values of features such as the TCA cycle, fatty acid synthesis, nucleotide metabolism, and branched-chain amino acid metabolism decrease the probability of predicting extreme hypoxia.

### 3.3 Systems-level flux analysis of top SHAP-ranked metabolic features

Reaction-level trajectory plots were generated for the top 12 SHAP-ranked metabolic features with an absolute dominant-bin SHAP value of ≥ 0.02 across any oxygen state (shown in Figures 4A–L).

1. ROS detoxification: Reaction-level trajectory analysis identified superoxide dismutase (peroxisomal reactions), catalase, and hydrogen peroxide oxidoreductase as the most divergent reactions in terms of fluxes across 10 oxygen fractions (Figure 4A). We observe a decreasing trend in the activities of all superoxide dismutase reactions from normoxia to extreme hypoxia. Hydrogen peroxide oxidoreductase has a peak at a normoxic and a hypoxic fraction. Catalase has a peak at an extreme hypoxic fraction.
2. Transport, mitochondrial: Reaction-level trajectory analysis revealed progressive suppression of transport of citrate and pyruvate, the aspartate-glutamate shuttle, malate, and ornithine mitochondrial transport from normoxia to decreasing oxygen gradients (Figure 4B).
3. Fatty acid synthesis: We observe changes in reactions such as stearoyl-CoA 9-desaturase and various fatty-acid synthase reactions, which are the dominant contributors to the dysregulation of the fatty acid synthesis pathway (Figure 4C). Changes in directionality between normoxic and extreme hypoxic regions reveal differences in NAD(P)H consumption in the latter.
4. ROS demand total: Two reactive oxygen species, hydrogen peroxide and hydroxyl radical, constitute the total ROS demand in our models, which shows that hydrogen peroxide demand decreases gradually from normoxia to the hypoxic states (Figure 4D). Hydroxyl radical demand sustains under hypoxia before declining under extreme hypoxia states.
5. Squalene and cholesterol synthesis: Trajectory analysis distinctly identified desmosterol reductase, isopentenyl-diphosphate D-isomerase, and cytosolic phosphomevalonate kinase as the primary contributors (Figure 4E). Desmosterol reductase shows a slight upward trend under hypoxia and extreme hypoxia. The remaining two reactions have a constant trend, with a slight decrease in extreme hypoxia.
6. Transport, endoplasmic reticular: Reaction-level trajectory analysis of cholesterol transport, lipid flip-flop transport, ceramide transport, fatty acid intracellular transport, and ER oxygen transport revealed mixed trends across the 3 oxygen gradients (Figure 4F). Fatty acid and ceramide remain almost stable across the three states. Cholesterol transport decreases in magnitude (approaching zero) and reverses direction at extreme hypoxia. Lipid flip-flop intracellular transport increases from normoxia to hypoxia, then drastically reduces from the later hypoxic region into extreme hypoxia, changes direction, and becomes stable thereafter.
7. Nucleotide metabolism: Nucleotide metabolism showed a sharp decrease in hypoxia and extreme hypoxia compared to normoxia, driven mainly by suppression of nucleoside-triphosphate diphosphatase and DNA-directed DNA polymerase reactions (Figure 4G).
8. Vitamin A metabolism: Trajectory analysis revealed dynamic trends of reactions like retinal dehydrogenase, 13-cis-retinoic acid isomerase, and multiple retinol dehydrogenase reactions (Figure 4H). One of the retinol dehydrogenase reactions had reversed directionality, maintained across all three states. All these reactions have peaks at at least one fraction of the 3 states.
9. Citric acid cycle: Reaction-level trajectory analysis identified succinate-CoA ligase (ADP-forming), succinate dehydrogenase, and succinate-CoA ligase (GDP-forming) as the most variable reactions, all showing flux reductions and changes in directionality from normoxia to extreme hypoxia (Figure 4I). The absolute value remains high at normoxia and becomes near zero for hypoxia and extreme hypoxia states.
10. Mitochondrial RSS demand: The trajectory plot shows that mitochondrial hydrogen sulfide remains constant at normoxia, reduces at hypoxic fractions, and starts increasing at the extreme hypoxic fractions (Figure 4J).
11. Tryptophan metabolism: Trajectory analysis identified mitochondrial and peroxisomal 3-hydroxyacyl coenzyme A dehydratase, 2-oxoadipate shuttle, and mitochondrial and peroxisomal 3-hydroxyacyl coenzyme A dehydrogenase as the most variable reactions, and all of them have the same directionality in reverse directions (Figure 4K). 2-Oxoadipate Shuttle has a dip in the reverse direction at hypoxia, and all three reactions remain almost constant at normoxia, but with variations at different hypoxic and extreme hypoxia states.
12. PPP/glycolysis: Log ratio of pentose phosphate pathway/glycolysis depicts the fraction of glucose carbon that goes into PPP (Figure 4L). PPP branching increases from normoxia to hypoxia. It then decreases in hypoxic states, and further increases and saturates at extreme hypoxic states.

## 4 Discussion

Our pFBA flux results provide only indispensable metabolic pathways for pediatric cancers under the given hypoxic and minimal essential media constraints, without accounting for alternative pathways. This eliminates redundant or non-essential pathways in our analysis. pFBA values are advantageous in qualitative analysis of active reactions and pathway usage. We converted the pFBA fluxes of reactions into allocation-based and ratios-based pathway features to capture meaningful cancer metabolic strategies. From the differences in colors across the three labels in Figure 3, we are able to infer metabolic pathways that the ML classifier uses to classify each oxygenated state. It explains which normalized pathway fractions and pathway ratios are used by the ML classifiers in distinguishing the three oxygen states. To further understand how metabolism changes as cancer cells progress from normoxia to extreme hypoxia, we plotted the trajectories of original signed flux values of the most divergent reactions belonging to the discriminative features from SHAP analysis (Figure 4).

- Redox imbalance: Features with high mean SHAP values are ROS detoxification and total ROS demand (constituted by mitochondrial and peroxisomal hydrogen peroxide, and cytosolic hydroxyl radical) (Figure 3A). The reduced fluxes of reactions under ROS detoxification in extreme hypoxia from the trajectory plot (Figure 4A), along with its highest positive SHAP value from the lower regime dominant bin of 0.11 (Figure 3B), make this the most discriminative metabolic signature that is negatively associated with extreme hypoxic conditions in pediatric cancers. We see that mitochondrial hydrogen sulfide demand flux increases under extreme hypoxic fractions, and also is positively associated with it in classification based on SHAP plot, as seen in Figures 3A,B,4J, [Fig F4] respectively. On the other hand, low values of total ROS demand increase the probability of predicting extreme hypoxia. So, it can be inferred that *RSS* > *ROS* in extreme hypoxia. Hydrogen sulfide is known to alleviate the damaging effects of ROS in hypoxia (Lan et al., 2011). It also has a therapeutic role in pediatric hematologic malignancies and other cancers (Lou et al., 2024; Iciek et al., 2023). Mitochondrial RSS demand is negatively associated with hypoxia prediction. An increase in total ROS demand is associated with a higher probability of hypoxia prediction, also hydroxyl radical sustains for most part of hypoxic state before reducing, as seen in Figure 4D. We observe that hypoxia classification is positively associated with high ROS detoxification, as evident from the positive SHAP value from the high feature bin (3A and B). Redox homeostasis in cancer cells protects mitochondrial reactive oxygen (ROS)-induced damage during hypoxia (Li et al., 2016). We infer that *ROS* > *RSS* in hypoxia. Normoxia classification is also positively associated with ROS detoxification, and only with ROS demand, not mitochondrial RSS.
- Oxygen-dependent pathways: Fatty acid oxidation, nucleotide metabolism, mitochondrial - cytosol transport, citric acid cycle, tryptophan and valine, leucine, and isoleucine metabolism increase the probability of normoxia prediction, but not the other two oxygen-deplete states. The mitochondrial transport feature has the second-highest mean SHAP value overall (Figure 3A). Trajectory plots reveal that citrate and malate are exported from mitochondria to the cytosol under normoxic conditions (Figure 4B). This also explains high activity in the citric acid cycle in normoxia (Figure 4I. It is known that citric acid cycle metabolites, amino acids, and fatty acids are transported to and from mitochondria during cancers (Chattopadhyay and Roy, 2017). Adult and pediatric cancers rely on fatty acid oxidation for energy, and this process is oxygen-dependent (Zhang, 2015; Salmanpour et al., 2025). The trajectory plot also revealed high flux through nucleotide metabolic enzyme (Figure 4G). Nucleotide metabolism encodes reactions that regulate the nucleotide pool and DNA repair. Cancer cells depend on DNA polymerases to sustain DNA damage under stress conditions (Barbari et al., 2022; Lange et al., 2011). Serotonin degradation enzymes branching from the tryptophan pathway have high flux differences from normoxia to extreme hypoxia relative to the commonly studied kynurenine pathway (Figure 4K). Serotonin degradation is reduced in the other two states compared to normoxia. We know that serotonin can contribute to tumor progression (Gautam et al., 2016). Metabolism of branched-chain amino acids is involved in tumor progression and treatment resistance (Zhou et al., 2025). A study contrasting our findings revealed that hypoxia induces enzymes involved in the metabolism of these amino acids (Zhang et al., 2021).
- Hypoxia-specific reprogramming: Cholesterol metabolism and synthesis are the two pathways that increase the probability of hypoxia prediction, but not the other two states, Figures 3A,B. Cholesterol biosynthesis is a metabolic vulnerability in pediatric tumors (Cousins et al., 2022; Gizaw et al., 2025). Pediatric tumors rely on cholesterol metabolism for proliferation, mediate signaling pathways, and overcome immune suppression (Zhang et al., 2025). Fatty acid synthesis in hypoxia shows a higher mean SHAP value than in normoxia (Figure 3A). Increased fatty acid synthesis seen under hypoxia compared to normoxia has been observed in some cancers (colorectal and breast cancers) where fatty acid synthesis is induced under hypoxia (Valli et al., 2014; Furuta et al., 2008). Fatty acid synthesis is an important hallmark of pediatric cancers (Salmanpour et al., 2025). Trajectory plot shows that lipid flip-flop has a huge peak at hypoxia (Figure 4C). This suggests that lipid metabolism reprogramming could be a potential target in hypoxia.
- Stress adaptive reprogramming: Pathways that indicate late-stage reprogramming to cope with oxygen depletion are transport, endoplasmic reticulum, glutaminolysis-driven TCA anaplerosis, and PPP branching from glycolysis. Oxygen import to ER slightly increases at extreme hypoxia to maintain oxygen levels required for sustenance, as shown in the trajectory plot (Figure 4F). ER undergoes oxidative stress under hypoxic conditions, which causes leakage of ROS outside (Arfin et al., 2021). Tumors get addicted to glutamine to supply substrates for the TCA cycle under hypoxia in pancreatic and lung tumors Ren et al. (2023); Vanhove et al. (2019). Glutamine addiction is also seen in pediatric cancers (Salmanpour et al., 2025). It is generally observed that cancer cells upregulate glycolysis for energy production (Sviderskiy et al., 2025). A study showed that hypoxia induces branching from glycolysis to PPP Cheung et al. (2012). As seen in Figures 3A,B, we observe PPP/glycolysis is positively associated with extreme hypoxia but not normoxia. Trajectory plots on PPP branching from glycolysis in the Supplementary Figure S5 show a mild increase in the ratio of PPP branching from glycolysis reactions at extreme hypoxic conditions compared to hypoxia (Figure 4L).
- Metabolic rewiring under oxygen deficiency: A feature that increases the probability of predicting the two oxygen-deplete states, but not normoxia, is vitamin A metabolism, seen in (Figures 3A,B). Trajectory analysis also revealed activation at different points under hypoxic and extreme hypoxic fractions (Figure 4H). *In vitro* studies on neuroblastoma (the largest cancer group in our cell lines) reveal that hypoxia induces retinoic acid pathways in neuroblastoma, which are responsible for cancer cell differentiation and proliferation (Brum et al., 2022).

In summary, we successfully applied machine learning algorithms on parsimonious flux analysis data to distinguish between normoxic, hypoxic, and extreme hypoxic states of ten pediatric cancers. We converted it into a multi-class classification problem (extreme hypoxia, hypoxia, and normoxia) and used pFBA flux-derived features to classify the three states. We then performed a systems-level metabolic flux analysis on the most dynamic reactions whose fluxes differed between normoxic and extreme-hypoxic states. Metabolic consistency correlation analysis revealed that these cancers represent a globally conserved metabolic signature. Research on comprehensive metabolic rewiring in pediatric cancers in hypoxic environments is sparse. Our work has established that pediatric cancers represent a distinct reactive species metabolic signature at hypoxic and extreme hypoxic conditions. Our findings reveal that pediatric cancers depend on reactive sulfur species to counteract oxidative stress during extreme hypoxia, and a hypoxic state is characterized by heightened ROS detoxification to counteract ROS. Integration of multi-omics data (proteomics and metabolomics) from pediatric cancers under hypoxic conditions to reconstruct models and as constraints will further improve metabolic modeling predictions. Such context-specific data, when they become available, can be used to validate our predictions.

## Data Availability

The codes used in the modification and analysis of GEMs, feature generation, and ML analysis are provided in the following repository https://doi.org/10.5281/zenodo.18549271. Further inquiries can be directed to the corresponding authors.

## References

[B1] Antonakoudis A. Barbosa R. Kotidis P. Kontoravdi C. (2020). The era of big data: genome-scale modelling meets machine learning. Comput. Struct. Biotechnol. J. 18, 3287–3300. 10.1016/j.csbj.2020.10.011 33240470 PMC7663219

[B2] Arafeh R. Shibue T. Dempster J. M. Hahn W. C. Vazquez F. (2025). The present and future of the cancer dependency map. Nat. Rev. Cancer 25, 59–73. 10.1038/s41568-024-00763-x 39468210

[B3] Arfin S. Jha N. K. Jha S. K. Kesari K. K. Ruokolainen J. Roychoudhury S. (2021). Oxidative stress in cancer cell metabolism. Antioxidants 10, 642. 10.3390/antiox10050642 33922139 PMC8143540

[B4] Barbari S. R. Beach A. K. Markgren J. G. Parkash V. Moore E. A. Johansson E. (2022). Enhanced polymerase activity permits efficient synthesis by cancer-associated dna polymerase *ϵ* variants at low dntp levels. Nucleic Acids Research 50, 8023–8040. 10.1093/nar/gkac602 35822874 PMC9371911

[B5] Bradley A. P. (1997). The use of the area under the roc curve in the evaluation of machine learning algorithms. Pattern Recognition 30, 1145–1159. 10.1016/s0031-3203(96)00142-2

[B6] Brum P. O. Viola G. D. Saibro-Girardi C. Tiefensee-Ribeiro C. Brum M. O. Gasparotto J. (2022). Hypoxia-inducible factor-1*α* (hif-1*α*) inhibition impairs retinoic acid-induced differentiation in sh-sy5y neuroblastoma cells, leading to reduced neurite length and diminished gene expression related to cell differentiation. Neurochem. Res. 47, 409–421. 10.1007/s11064-021-03454-3 34557995 PMC8827409

[B7] Brunk E. Sahoo S. Zielinski D. C. Altunkaya A. Dräger A. Mih N. (2018). Recon3d enables a three-dimensional view of gene variation in human metabolism. Nat. Biotechnology 36, 272–281. 10.1038/nbt.4072 29457794 PMC5840010

[B8] Çakr T. Alsan S. Saybaş H. Akn A. Ülgen K. Ö. (2007). Reconstruction and flux analysis of coupling between metabolic pathways of astrocytes and neurons: application to cerebral hypoxia. Theor. Biol. Med. Model. 4, 48. 10.1186/1742-4682-4-48 18070347 PMC2246127

[B9] Chattopadhyay E. Roy B. (2017). Altered mitochondrial signalling and metabolism in cancer. Front. Oncology 7, 43. 10.3389/fonc.2017.00043 28373964 PMC5357656

[B10] Chen X. Yang W. Roberts C. W. Zhang J. (2024). Developmental origins shape the paediatric cancer genome. Nat. Rev. Cancer 24, 382–398. 10.1038/s41568-024-00684-9 38698126 PMC11571274

[B11] Cheung E. C. Ludwig R. L. Vousden K. H. (2012). Mitochondrial localization of tigar under hypoxia stimulates hk2 and lowers ros and cell death. Proc. Natl. Acad. Sci. 109, 20491–20496. 10.1073/pnas.1206530109 23185017 PMC3528527

[B12] Chung C. H. Chandrasekaran S. (2022). A flux-based machine learning model to simulate the impact of pathogen metabolic heterogeneity on drug interactions. PNAS Nexus 1, pgac132. 10.1093/pnasnexus/pgac132 36016709 PMC9396445

[B13] Cousins A. Olivares O. Markert E. Manoharan A. Bubnova X. Bresolin S. (2022). Central nervous system involvement in childhood acute lymphoblastic leukemia is linked to upregulation of cholesterol biosynthetic pathways. Leukemia 36, 2903–2907. 10.1038/s41375-022-01722-x 36289348 PMC9712090

[B14] Czajka J. J. Oyetunde T. Tang Y. J. (2021). Integrated knowledge mining, genome-scale modeling, and machine learning for predicting yarrowia lipolytica bioproduction. Metab. Eng. 67, 227–236. 10.1016/j.ymben.2021.07.003 34242777

[B15] Dai Z. Yang S. Xu L. Hu H. Liao K. Wang J. (2019). Identification of cancer–associated metabolic vulnerabilities by modeling multi-objective optimality in metabolism. Cell Commun. Signal. 17, 1–15. 10.1186/s12964-019-0439-y 31601242 PMC6785927

[B16] Damiani C. Maspero D. Di Filippo M. Colombo R. Pescini D. Graudenzi A. (2019). Integration of single-cell rna-seq data into population models to characterize cancer metabolism. PLoS Computational Biology 15, e1006733. 10.1371/journal.pcbi.1006733 30818329 PMC6413955

[B17] Dharia N. V. Kugener G. Guenther L. M. Malone C. F. Durbin A. D. Hong A. L. (2021). A first-generation pediatric cancer dependency map. Nat. Genetics 53, 529–538. 10.1038/s41588-021-00819-w 33753930 PMC8049517

[B18] Eames A. Chandrasekaran S. (2024). Leveraging metabolic modeling and machine learning to uncover modulators of quiescence depth. PNAS Nexus 3, pgae013. 10.1093/pnasnexus/pgae013 38292544 PMC10825626

[B19] Ebrahim A. Lerman J. A. Palsson B. O. Hyduke D. R. (2013). Cobrapy: constraints-based reconstruction and analysis for python. BMC Systems Biology 7, 74. 10.1186/1752-0509-7-74 23927696 PMC3751080

[B20] Eyassu F. Angione C. (2017). Modelling pyruvate dehydrogenase under hypoxia and its role in cancer metabolism. R. Soc. Open Science 4, 170360. 10.1098/rsos.170360 29134060 PMC5666243

[B21] Furuta E. Pai S. K. Zhan R. Bandyopadhyay S. Watabe M. Mo Y.-Y. (2008). Fatty acid synthase gene is up-regulated by hypoxia via activation of akt and sterol regulatory element binding protein-1. Cancer Research 68, 1003–1011. 10.1158/0008-5472.CAN-07-2489 18281474

[B22] Gautam J. Banskota S. Regmi S. C. Ahn S. Jeon Y. H. Jeong H. (2016). Tryptophan hydroxylase 1 and 5-ht7 receptor preferentially expressed in triple-negative breast cancer promote cancer progression through autocrine serotonin signaling. Mol. Cancer 15, 75. 10.1186/s12943-016-0559-6 27871326 PMC5117586

[B23] Gizaw N. Y. Kolari K. Kallio P. Alitalo K. Kivelä R. (2025). Inhibiting cholesterol synthesis halts rhabdomyosarcoma growth via er stress and cell cycle arrest. EMBO Mol. Med. 17, 3586–3606. 10.1038/s44321-025-00336-x 41249736 PMC12686467

[B24] Gröbner S. N. Worst B. C. Weischenfeldt J. Buchhalter I. Kleinheinz K. Rudneva V. A. (2018). The landscape of genomic alterations across childhood cancers. Nature 555, 321–327. 10.1038/nature25480 29489754

[B25] Hand D. J. Till R. J. (2001). A simple generalisation of the area under the roc curve for multiple class classification problems. Mach. Learning 45, 171–186. 10.1023/a:1010920819831

[B26] Heirendt L. Arreckx S. Pfau T. Mendoza S. N. Richelle A. Heinken A. (2019). Creation and analysis of biochemical constraint-based models using the cobra toolbox v. 3.0. Nat. Protocols 14, 639–702. 10.1038/s41596-018-0098-2 30787451 PMC6635304

[B27] Hompland T. Fjeldbo C. S. Lyng H. (2021). Tumor hypoxia as a barrier in cancer therapy: why levels matter. Cancers 13, 499. 10.3390/cancers13030499 33525508 PMC7866096

[B28] Iciek M. Bilska-Wilkosz A. Kozdrowicki M. Górny M. (2023). Reactive sulfur species in human diseases. Antioxidants and Redox Signal. 39, 1000–1023. 10.1089/ars.2023.0261 37440317

[B29] Issaq S. H. Heske C. M. (2020). Targeting metabolic dependencies in pediatric cancer. Curr. Opinion Pediatrics 32, 26–34. 10.1097/MOP.0000000000000853 31789976 PMC7263318

[B30] Kumar S. P. Bhatt N. P. (2025). Modelling reliable metabolic phenotypes by analysing the context-specific transcriptomics data. Npj Syst. Biol. Appl. 11, 142. 10.1038/s41540-025-00617-8 41390530 PMC12739174

[B62] Kumar S. P. Sridhar S. Alsmadi N. Mahadevan R. Bhatt N. (2026). Generalist method to reconstruct metabolic networks from multi-omics data at large-scale. bioRxiv. 10.64898/2026.04.02.716249

[B31] Lan A. Liao X. Mo L. Yang C. Yang Z. Wang X. (2011). Hydrogen sulfide protects against chemical hypoxia-induced injury by inhibiting ros-activated erk1/2 and p38mapk signaling pathways in pc12 cells. PloS One 6, e25921. 10.1371/journal.pone.0025921 21998720 PMC3187826

[B32] Lange S. S. Takata K.-i. Wood R. D. (2011). Dna polymerases and cancer. Nat. Reviews Cancer 11, 96–110. 10.1038/nrc2998 21258395 PMC3739438

[B33] Lewis J. E. Kemp M. L. (2021). Integration of machine learning and genome-scale metabolic modeling identifies multi-omics biomarkers for radiation resistance. Nat. Communications 12, 2700. 10.1038/s41467-021-22989-1 33976213 PMC8113601

[B34] Lewis N. E. Hixson K. K. Conrad T. M. Lerman J. A. Charusanti P. Polpitiya A. D. (2010). Omic data from evolved e. coli are consistent with computed optimal growth from genome-scale models. Mol. Syst. Biol. 6, 390. 10.1038/msb.2010.47 20664636 PMC2925526

[B35] Li P. Zhang D. Shen L. Dong K. Wu M. Ou Z. (2016). Redox homeostasis protects mitochondria through accelerating ros conversion to enhance hypoxia resistance in cancer cells. Sci. Reports 6, 22831. 10.1038/srep22831 26956544 PMC4783784

[B36] Lou S. Jiang Z.-L. Zhu Y.-W. Zhang R.-Y. Wang Y. Chu T. (2024). Exploring the impact of hydrogen sulfide on hematologic malignancies: a review. Cell. Signal. 120, 111236. 10.1016/j.cellsig.2024.111236 38810860

[B37] Lundberg S. M. Lee S.-I. (2017). A unified approach to interpreting model predictions. Adv. Neural Information Processing Systems 30, 4768–4777. 10.5555/3295222.3295230

[B38] Omer S. Sridhar S. Karunagaran D. Suraishkumar G. (2025). Mechanistic insights into hypoxia-induced metabolic reprogramming in colorectal cancer through genome-scale modeling. Biotechnol. Prog. 41, e70002. 10.1002/btpr.70002 39964193

[B39] Orth J. D. Thiele I. Palsson B. Ø. (2010). What is flux balance analysis? Nat. Biotechnology 28, 245–248. 10.1038/nbt.1614 20212490 PMC3108565

[B40] Pedregosa F. Varoquaux G. Gramfort A. Michel V. Thirion B. Grisel O. (2011). Scikit-learn: machine learning in python. The J. Machine Learn. Research 12, 2825–2830.

[B41] Razmpour T. Tabibian M. Roohi A. Saha R. (2026). Gan-enhanced machine learning and metabolic modeling identify reprogramming in pancreatic cancer. PLOS Comput. Biol. 22, e1013862. 10.1371/journal.pcbi.1013862 41481753 PMC12779136

[B42] Ren L.-L. Mao T. Meng P. Zhang L. Wei H.-Y. Tian Z.-B. (2023). Glutamine addiction and therapeutic strategies in pancreatic cancer. World J. Gastrointest. Oncol. 15, 1852–1863. 10.4251/wjgo.v15.i11.1852 38077649 PMC10701242

[B43] Salmanpour F. Alijanzadeh D. Ghobadinezhad F. Samieefar N. Piryaee M. Hosseini Bajestani Z. (2025). “Metabolic pathways in pediatric cancers: current findings and targets for therapies,” in Novel approaches in cancer treatment: tumor targeted therapy (Springer), 913–941.

[B44] Sokolova M. Lapalme G. (2009). A systematic analysis of performance measures for classification tasks. Inf. Process. Manag. 45, 427–437. 10.1016/j.ipm.2009.03.002

[B45] Sridhar S. Bhalla P. Kullu J. Veerapaneni S. Sahoo S. Bhatt N. (2023). A reactive species reactions module for integration into genome-scale metabolic models for improved insights: application to cancer. Metab. Eng. 80, 78–93. 10.1016/j.ymben.2023.08.006 37689259

[B46] Sun C. X. Daniel P. Bradshaw G. Shi H. Loi M. Chew N. (2023). Generation and multi-dimensional profiling of a childhood cancer cell line atlas defines new therapeutic opportunities. Cancer Cell 41, 660–677. 10.1016/j.ccell.2023.03.007 37001527

[B47] Sviderskiy V. O. Vasudevaraja V. Dubois L. G. Stafford J. Liu E. K. Serrano J. (2025). Metabolic profiling of adult and pediatric gliomas reveals enriched glucose availability in pediatric gliomas and increased fatty acid oxidation in adult gliomas. Acta Neuropathol. Commun. 13, 61. 10.1186/s40478-025-01961-w 40087788 PMC11909955

[B48] Takahashi E. Yamaoka Y. (2017). Simple and inexpensive technique for measuring oxygen consumption rate in adherent cultured cells. J. Physiol. Sci. 67, 731–737. 10.1007/s12576-017-0563-7 28785888 PMC10717709

[B49] Tavakoli N. Fong E. J. Coleman A. Huang Y.-K. Bigger M. Doche M. E. (2025). Merging metabolic modeling and imaging for screening therapeutic targets in colorectal cancer. NPJ Systems Biology Applications 11, 12. 10.1038/s41540-025-00494-1 39875420 PMC11775273

[B50] Valli A. Rodriguez M. Moutsianas L. Fischer R. Fedele V. Huang H.-L. (2014). Hypoxia induces a lipogenic cancer cell phenotype via hif1*α*-dependent and-independent pathways. Oncotarget 6, 1920–1941. 10.18632/oncotarget.3058 25605240 PMC4385826

[B51] Vanhove K. Derveaux E. Graulus G.-J. Mesotten L. Thomeer M. Noben J.-P. (2019). Glutamine addiction and therapeutic strategies in lung cancer. Int. Journal Molecular Sciences 20, 252. 10.3390/ijms20020252 30634602 PMC6359540

[B52] Vijayakumar S. Magazzù G. Moon P. Occhipinti A. Angione C. (2022). “A practical guide to integrating multimodal machine learning and metabolic modeling,” in Computational systems biology in medicine and biotechnology: methods and protocols (Springer), 87–122. 10.1007/978-1-0716-1831-8_5 35604554

[B53] Wilson K. A. Talty C.-E. Parker B. C. VandeVord P. J. (2025). Integrative constraint-based modeling and proteomics uncover astrocytic metabolic adaptations to the post-tbi microenvironment. Int. J. Mol. Sci. 26, 6456. 10.3390/ijms26136456 40650231 PMC12249836

[B54] Woo H. Kim Y. Kim D. Yoon S. H. (2024). Machine learning identifies key metabolic reactions in bacterial growth on different carbon sources. Mol. Syst. Biol. 20, 170–186. 10.1038/s44320-024-00017-w 38291231 PMC10912204

[B55] Yang G. Huang S. Hu K. Lu A. Yang J. Meroueh N. (2023). Flux estimation analysis systematically characterizes the metabolic shifts of the central metabolism pathway in human cancer. Front. Oncol. 13, 1117810. 10.3389/fonc.2023.1117810 37377905 PMC10291142

[B56] Zampieri G. Vijayakumar S. Yaneske E. Angione C. (2019). Machine and deep learning meet genome-scale metabolic modeling. PLoS Comput. Biol. 15, e1007084. 10.1371/journal.pcbi.1007084 31295267 PMC6622478

[B57] Zehetner L. Széliová D. Kraus B. Hernandez Bort J. A. Zanghellini J. (2024). Logistic pca explains differences between genome-scale metabolic models in terms of metabolic pathways. PLOS Comput. Biol. 20, e1012236. 10.1371/journal.pcbi.1012236 38913731 PMC11226097

[B58] Zhang H. (2015). Hif-1 suppresses lipid catabolism to promote cancer progression. Mol. and Cellular Oncology 2, e980184. 10.4161/23723556.2014.980184 27308514 PMC4905416

[B59] Zhang B. Chen Y. Shi X. Zhou M. Bao L. Hatanpaa K. J. (2021). Regulation of branched-chain amino acid metabolism by hypoxia-inducible factor in glioblastoma. Cell. Mol. Life Sci. 78, 195–206. 10.1007/s00018-020-03483-1 32088728 PMC8112551

[B60] Zhang W. Xu Y. Fang Y. Li M. Li D. Guo H. (2025). Ubiquitination in lipid metabolism reprogramming: implications for pediatric solid tumors. Front. Immunol. 16, 1554311. 10.3389/fimmu.2025.1554311 40370434 PMC12075147

[B61] Zhou Y. Kou J. Li W. Wang Y. Su X. Zhang H. (2025). Bcaa metabolism in cancer progression and therapy resistance: the balance between fuel and cell signaling. Front. Pharmacol. 16, 1595176. 10.3389/fphar.2025.1595176 40438606 PMC12116492

