# Supplementary material for: Machine learning and metabolic modeling-based identification of hypoxia-driven metabolic signatures in pediatric cancers
Source: Front Pharmacol. 2026 Apr 30;17:1810370. doi: 10.3389/fphar.2026.1810370 (PMC13171815; doi:10.3389/fphar.2026.1810370)
Supplement: Supplementary file 1 [file DataSheet1.pdf]

frontiers

Supplementary Material

1 SUPPLEMENTARY TABLES

We have provided the details of the pediatric cancer cell lines used in the reconstruction of metabolic models (Kumar S et al. (2026)) in Table S1.

**Model ID**	**Cell-line Name**	**Cancer Type**	**Resource Identifier**	**Source**
ACH-000080	BDCM	Acute Myeloid Leukemia	CVCL_4613	ATCC
ACH-000045	MV4-11	Acute Myeloid Leukemia	CVCL_0064	ATCC
ACH-000146	THP-1	Acute Myeloid Leukemia	CVCL_0006	ATCC
ACH-000263	KASUMI-1	Acute Myeloid Leukemia	CVCL_0589	DSMZ
ACH-000602	M-07e	Acute Myeloid Leukemia	CVCL_2106	DSMZ
ACH-000770	P31/FUJ	Acute Myeloid Leukemia	CVCL_1632	HSRRB
ACH-000557	AML-193	Acute Myeloid Leukemia	CVCL_1071	ATCC
ACH-000641	CMK	Acute Myeloid Leukemia	CVCL_0216	DSMZ
ACH-001036	CMK-11-5	Acute Myeloid Leukemia	CVCL_0217	HSRRB
ACH-000020	MHH-CALL-2	B-Cell Acute Lymphoblastic Leukemia	CVCL_1409	DSMZ
ACH-000032	MHH-CALL-3	B-Cell Acute Lymphoblastic Leukemia	CVCL_0089	DSMZ
ACH-000059	SUP-B15	B-Cell Acute Lymphoblastic Leukemia	CVCL_0103	ATCC
ACH-000070	697	B-Cell Acute Lymphoblastic Leukemia	CVCL_0079	DSMZ
ACH-000151	JM1	B-Cell Acute Lymphoblastic Leukemia	CVCL_3532	ATCC
ACH-000156	MHH-CALL-4	B-Cell Acute Lymphoblastic Leukemia	CVCL_1410	DSMZ
ACH-000728	KASUMI-2	B-Cell Acute Lymphoblastic Leukemia	CVCL_0590	DSMZ
ACH-000782	SEM	B-Cell Acute Lymphoblastic Leukemia	CVCL_0095	DSMZ
ACH-000922	RCH-ACV	B-Cell Acute Lymphoblastic Leukemia	CVCL_1851	DSMZ
ACH-000960	Reh	B-Cell Acute Lymphoblastic Leukemia	CVCL_1650	ATCC
ACH-001106	KOPN-8	B-Cell Acute Lymphoblastic Leukemia	CVCL_1866	DSMZ
ACH-001669	TANOUE	B-Cell Acute Lymphoblastic Leukemia	CVCL_1852	DSMZ

ACH-001735	SEMK2	B-Cell Acute Lymphoblastic Leukemia	CVCL_S906	Dana-Farber Cancer Institute
ACH-001736	HB1119	B-Cell Acute Lymphoblastic Leukemia	CVCL_8227	Dana-Farber Cancer Institute
ACH-001993	NALM-16	B-Cell Acute Lymphoblastic Leukemia	CVCL_1834	DSMZ
ACH-002059	P30/OHK	B-Cell Acute Lymphoblastic Leukemia	CVCL_1631	RIKEN
ACH-000160	BT-12	Embryonal Tumor	CVCL_M155	St. Jude Children's Research Hospital
ACH-001020	BT-16	Embryonal Tumor	CVCL_M156	St. Jude Children's Research Hospital
ACH-001028	CHLA-06-ATRT	Embryonal Tumor	CVCL_AQ42	ATCC
ACH-001031	CHLA-266	Embryonal Tumor	CVCL_M149	Children's Oncology Group (COG)
ACH-001289	COG-AR-359	Embryonal Tumor	CVCL_RS25	Children's Oncology Group (COG)
ACH-000055	D283 Med	Embryonal Tumor	CVCL_1155	ATCC
ACH-000095	D341 Med	Embryonal Tumor	CVCL_0018	ATCC
ACH-000211	Daoy	Embryonal Tumor	CVCL_1167	ATCC
ACH-000776	ONS-76	Embryonal Tumor	CVCL_1624	HSRRB
ACH-001053	D425	Embryonal Tumor	CVCL_1275	Ohio State University
ACH-001054	D458	Embryonal Tumor	CVCL_1161	Ohio State University
ACH-001201	SU-MB-002	Embryonal Tumor	CVCL_VU79	Ohio State University
ACH-001232	UW228	Embryonal Tumor	CVCL_8585	Dana-Farber Cancer Institute
ACH-001033	CHLA-57	Embryonal Tumor	CVCL_0B49	Children's Oncology Group (COG)
ACH-001711	PFSK-1	Embryonal Tumor	CVCL_1642	ATCC
ACH-000039	SK-N-MC	Ewing Sarcoma	CVCL_0530	ATCC

ACH-000052	A-673	Ewing Sarcoma	CVCL_0080	ATCC
ACH-000087	SK-ES-1	Ewing Sarcoma	CVCL_0627	ATCC
ACH-000279	EWS502	Ewing Sarcoma	CVCL_S740	Brigham and Women's Hospital
ACH-000391	MHH-ES-1	Ewing Sarcoma	CVCL_1411	DSMZ
ACH-000499	EW8	Ewing Sarcoma	CVCL_V618	Dana-Farber Cancer Institute
ACH-001029	CHLA-10	Ewing Sarcoma	CVCL_6583	Children's Oncology Group (COG)
ACH-001032	CHLA-32	Ewing Sarcoma	CVCL_M151	Children's Oncology Group (COG)
ACH-001034	CHLA-9	Ewing Sarcoma	CVCL_M150	Children's Oncology Group (COG)
ACH-001035	CHLA-99	Ewing Sarcoma	CVCL_0B50	Children's Oncology Group (COG)
ACH-001038	COG-E-352	Ewing Sarcoma	CVCL_M153	Children's Oncology Group (COG)
ACH-001193	SK-PN-DW	Ewing Sarcoma	CVCL_1703	Dana-Farber Cancer Institute
ACH-001430	TC-138	Ewing Sarcoma	CVCL_4Z30	Dana-Farber Cancer Institute
ACH-001431	TC-205	Ewing Sarcoma	CVCL_4Z31	Dana-Farber Cancer Institute
ACH-000245	BL-41	Mature B-Cell Neoplasms	CVCL_1087	DSMZ
ACH-000402	BL-70	Mature B-Cell Neoplasms	CVCL_1088	DSMZ
ACH-000707	P3HR-1	Mature B-Cell Neoplasms	CVCL_2676	ATCC
ACH-000786	Daudi	Mature B-Cell Neoplasms	CVCL_0008	ATCC
ACH-000877	EB1	Mature B-Cell Neoplasms	CVCL_2027	ATCC
ACH-000944	NAMALWA	Mature B-Cell Neoplasms	CVCL_0067	JCRB
ACH-001064	EB-2	Mature B-Cell Neoplasms	CVCL_1186	ATCC
ACH-002055	TL-1	Mature B-Cell Neoplasms	CVCL_B371	RIKEN
ACH-000660	SU-DHL-5	Mature B-Cell Neoplasms	CVCL_1735	DSMZ
ACH-000099	SIMA	Neuroblastoma	CVCL_1695	DSMZ
ACH-000120	CHP-212	Neuroblastoma	CVCL_1125	ATCC

ACH-000136	CHP-126	Neuroblastoma	CVCL_1123	DSMZ
ACH-000149	SK-N-SH	Neuroblastoma	CVCL_0531	ATCC
ACH-000203	NH-6	Neuroblastoma	CVCL_1606	HSRRB
ACH-000227	KP-N-YN	Neuroblastoma	CVCL_1341	HSRRB
ACH-000259	KELLY	Neuroblastoma	CVCL_2092	DSMZ
ACH-000260	SK-N-AS	Neuroblastoma	CVCL_1700	ATCC
ACH-000310	IMR-32	Neuroblastoma	CVCL_0346	ATCC
ACH-000312	SK-N-BE(2)	Neuroblastoma	CVCL_0528	ATCC
ACH-000341	SK-N-FI	Neuroblastoma	CVCL_1702	ATCC
ACH-000345	KP-N-RT-BM-1	Neuroblastoma	CVCL_1339	HSRRB
ACH-000366	SK-N-DZ	Neuroblastoma	CVCL_1701	ATCC
ACH-000446	KP-N-SI9s	Neuroblastoma	CVCL_1340	HSRRB
ACH-000804	NB-1	Neuroblastoma	CVCL_1440	HSRRB
ACH-001188	SH-SY5Y	Neuroblastoma	CVCL_0019	ATCC
ACH-001300	CHLA-15	Neuroblastoma	CVCL_6594	Children's Oncology Group (COG)
ACH-001301	COG-N-278	Neuroblastoma	CVCL_AX49	Children's Oncology Group (COG)
ACH-001302	COG-N-305	Neuroblastoma	CVCL_AX42	Children's Oncology Group (COG)
ACH-001303	NB-1643	Neuroblastoma	CVCL_5627	Broad Institute
ACH-001338	CHP-134	Neuroblastoma	CVCL_1124	Sigma-Aldrich
ACH-001344	GI-ME-N	Neuroblastoma	CVCL_1232	DSMZ
ACH-001354	LAN-2	Neuroblastoma	CVCL_1829	DSMZ
ACH-001366	NGP	Neuroblastoma	CVCL_2141	DSMZ
ACH-001367	NMB	Neuroblastoma	CVCL_2143	DSMZ
ACH-001481	CHLA-90	Neuroblastoma	CVCL_6610	Children's Oncology Group (COG)
ACH-001548	LS	Neuroblastoma	CVCL_2105	DSMZ
ACH-001603	NH-12	Neuroblastoma	CVCL_1605	JCRB
ACH-001674	TGW	Neuroblastoma	CVCL_1771	JCRB
ACH-001716	GOTO	Neuroblastoma	CVCL_1234	JCRB
ACH-002922	SK-N-MM	Neuroblastoma	CVCL_C8G1	Memorial Sloan Kettering Cancer Center

ACH-000082	G-292, clone A141B1	Osteosarcoma	CVCL_2909	ATCC
ACH-000359	MG-63	Osteosarcoma	CVCL_0426	ATCC
ACH-000364	U-2 OS	Osteosarcoma	CVCL_0042	ATCC
ACH-000410	Saos-2	Osteosarcoma	CVCL_0548	ATCC
ACH-000613	HOS	Osteosarcoma	CVCL_0312	ATCC
ACH-001001	143B	Osteosarcoma	CVCL_2270	ATCC
ACH-001526	HuO9	Osteosarcoma	CVCL_1298	JCRB
ACH-001715	CAL-72	Osteosarcoma	CVCL_1113	DSMZ
ACH-001814	OS252	Osteosarcoma	0	Montefiore Einstein
ACH-002067	NOS-1	Osteosarcoma	CVCL_1610	RIKEN
ACH-002069	HS-Os-1	Osteosarcoma	CVCL_8716	RIKEN
ACH-002471	OS052	Osteosarcoma	CVCL_C8FR	UCSF Helen Diller Family Comprehensive Cancer Center
ACH-002834	OS384	Osteosarcoma	CVCL_C8FU	UCSF Helen Diller Family Comprehensive Cancer Center
ACH-000597	TTC-709	Rhabdoid Cancer	CVCL_8007	St. Jude Children's Research Hospital
ACH-001059	DL	Rhabdoid Cancer	CVCL_U760	St. Jude Children's Research Hospital
ACH-001099	KD	Rhabdoid Cancer	CVCL_U757	St. Jude Children's Research Hospital
ACH-001128	MON	Rhabdoid Cancer	CVCL_M846	St. Jude Children's Research Hospital
ACH-001211	TTC-549	Rhabdoid Cancer	CVCL_8005	St. Jude Children's Research Hospital
ACH-000096	G-401	Rhabdoid Cancer	CVCL_0270	ATCC
ACH-000201	A-204	Rhabdoid Cancer	CVCL_1058	ATCC
ACH-000375	G-402	Rhabdoid Cancer	CVCL_1221	ATCC

ACH-000533	NCI-H2004 RT	Rhabdoid Cancer	CVCL_WS70	St. Jude Children's Research Hospital
ACH-000607	KYM-1	Rhabdoid Cancer	CVCL_3007	HSRRB
ACH-001109	KP-MRT-RY	Rhabdoid Cancer	CVCL_7051	St. Jude Children's Research Hospital
ACH-001200	STM91-01	Rhabdoid Cancer	CVCL_8000	St. Jude Children's Research Hospital
ACH-001210	TTC-1240	Rhabdoid Cancer	CVCL_8002	St. Jude Children's Research Hospital
ACH-001532	JMU-RTK-2	Rhabdoid Cancer	CVCL_4W58	JCRB
ACH-000833	RH-30	Rhabdomyosarcoma	CVCL_0041	DSMZ
ACH-001050	CW9019	Rhabdomyosarcoma	CVCL_N820	National Institutes of Health (NIH)
ACH-001096	JR	Rhabdomyosarcoma	CVCL_RT33	Broad Institute
ACH-001184	SCMC-RM2	Rhabdomyosarcoma	CVCL_A667	University Hospital Zurich
ACH-001740	RH28	Rhabdomyosarcoma	CVCL_8752	University Hospital Zurich
ACH-001743	RC2	Rhabdomyosarcoma	CVCL_L510	University Hospital Zurich
ACH-001745	RhJT	Rhabdomyosarcoma	CVCL_VU81	University Hospital Zurich
ACH-001765	Rh4	Rhabdomyosarcoma	CVCL_5916	Nationwide Children's Hospital
ACH-000169	RD	Rhabdomyosarcoma	CVCL_1649	ATCC
ACH-000689	RH-18	Rhabdomyosarcoma	CVCL_1659	DSMZ
ACH-001196	SMS-CTR	Rhabdomyosarcoma	CVCL_A770	Broad Institute
ACH-001750	TTC442	Rhabdomyosarcoma	CVCL_B255	University Hospital Zurich

ACH-001751	Rh36	Rhabdomyosarcoma	CVCL_M599	University Hospital Zurich
ACH-002048	RMS-YM	Rhabdomyosarcoma	CVCL_A792	RIKEN
ACH-000051	TE 617.T	Rhabdomyosarcoma	CVCL_1755	ATCC
ACH-000105	ALL-SIL	T-Lymphoblastic Leukemia/Lymphoma	CVCL_1805	DSMZ
ACH-000372	P12-ICHIKAWA	T-Lymphoblastic Leukemia/Lymphoma	CVCL_1630	DSMZ
ACH-000519	PEER	T-Lymphoblastic Leukemia/Lymphoma	CVCL_1913	HSRRB
ACH-000636	RPMI-8402	T-Lymphoblastic Leukemia/Lymphoma	CVCL_1667	DSMZ
ACH-000918	MOLT-16	T-Lymphoblastic Leukemia/Lymphoma	CVCL_1424	DSMZ
ACH-000937	PF-382	T-Lymphoblastic Leukemia/Lymphoma	CVCL_1641	DSMZ
ACH-000942	HPB-ALL	T-Lymphoblastic Leukemia/Lymphoma	CVCL_1820	DSMZ
ACH-000953	SUP-T1	T-Lymphoblastic Leukemia/Lymphoma	CVCL_1714	ATCC
ACH-000981	DND-41	T-Lymphoblastic Leukemia/Lymphoma	CVCL_2022	DSMZ
ACH-000995	JURKAT	T-Lymphoblastic Leukemia/Lymphoma	CVCL_0065	DSMZ
ACH-001737	CTV-1-DM	T-Lymphoblastic Leukemia/Lymphoma	CVCL_1150	Dana-Farber Cancer Institute

Table S1: Details of the 147 cancer cells taken from the Cancer Dependency Map

We have provided the number of pediatric cancer genome-scale metabolic models for each 10 cancer types in Table S2.

2 METABOLIC MODELS

We developed an in-silico experimental setup to convert the Basal Medium Eagle media (BME) to constraints. This was chosen to ensure a metabolically non-complex nutrient uptake constraint. The following assumptions were made to convert the BME media composition into the media constraints for the cancer genome-scale metabolic models(GEMs). The cancer cells were assumed to be in the exponential phase of their growth cycle in a petri plate with a capacity of 3 ml. For the cell line experiments, the total duration of the experimental period is assumed to be 5×24 hours (5 days) for cancer cells, and 10^6 cells are assumed to be present in the plate at the end of the experimental duration. Average biomass content

S.No	Cancers	Count
1	Acute Myeloid Leukemia	9
2	B-Cell Acute Lymphoblastic Leukemia	16
3	Embryonal Tumor	15
4	Ewing Sarcoma	14
5	Neuroblastoma	31
6	Osteosarcoma	13
7	Rhabdomyosarcoma	15
8	T-Lymphoblastic Leukemia/Lymphoma	11
9	Mature B-Cell Neoplasms	9
10	Rhabdoid Cancer	14

Table S2. Total number of genome-scale metabolic models in ten pediatric cancers

per cell, 264 pg dry weight/cell, was taken from Széliová et al. (2020). We converted the concentration of each metabolite of the BME into maximum allowable uptake bound by dividing them by the duration, cell numbers, and biomass content per cell, and multiplying by the petri plate capacity.

(i) BME media volume: $3ml(3 \times 10^{-3}\ l)$

(ii) Growth period of the cancer cells: 120 hours

(iii) Average content of biomass dry cell weight: 264 pg/cell

For cancer cells

$$\frac{\text{Concentration of each metabolite in BME media}(mmol\ l^{-1}) \times 3 \times 10^{-3}(l)}{120(h) \times 264 \times 10^{-12}(\text{gDW}/cell) \times 10^{6}(cell)}$$

Final units of exchange flux: $mmol\, gDW^{-1}\text{h}^{-1}$

3 METABOLIC CONSISTENCY OF PEDIATRIC CANCERS

Figure S1 shows the correlation coefficient ρ values of 10 pediatric cancers in a horizontal bar plot, color-coded by oxygen states. We measured the ratios of pathway fluxes to the total fluxes in every 147 GEM in all oxygen states. The total number of metabolic pathways that include reactive species demands is 99. The oxidative phosphorylation pathway was removed from the list of pathways because we constrained the reaction from this pathway to simulate the three oxygen states. We calculated a median value for all normalized metabolic pathway flux fractions in the 10 cancers for all three oxygen states. We then calculated a global mean value from the median pathway allocation ratios. We have added a baseline value of $\rho = 0.85$ in the plot, and we see that 6 out of 10 cancers have very high correlation coefficient values in all three oxygen states. All these 6 pediatric cancer categories have similar pathway allocation strategies compared to the common strategy observed in all 10 cancers. In the remaining 4 cancers, 3 cancers (osteosarcoma, rhabdomyosarcoma, and T-lymphoblastic leukemia/lymphoma) have $\rho < 0.85$ in only one oxygen state. Rhabdomyosarcoma has the lowest $\rho = 0.75$ of all cases, in extreme hypoxia. Mature B-Cell Neoplasms is the only cancer that has $\rho < 0.85$ in two oxygen states. It could be due to the

fact that these two cancers have distinct hypoxic micro-environments, respectively (Bernauer et al., 2021; Bhalla et al., 2018).

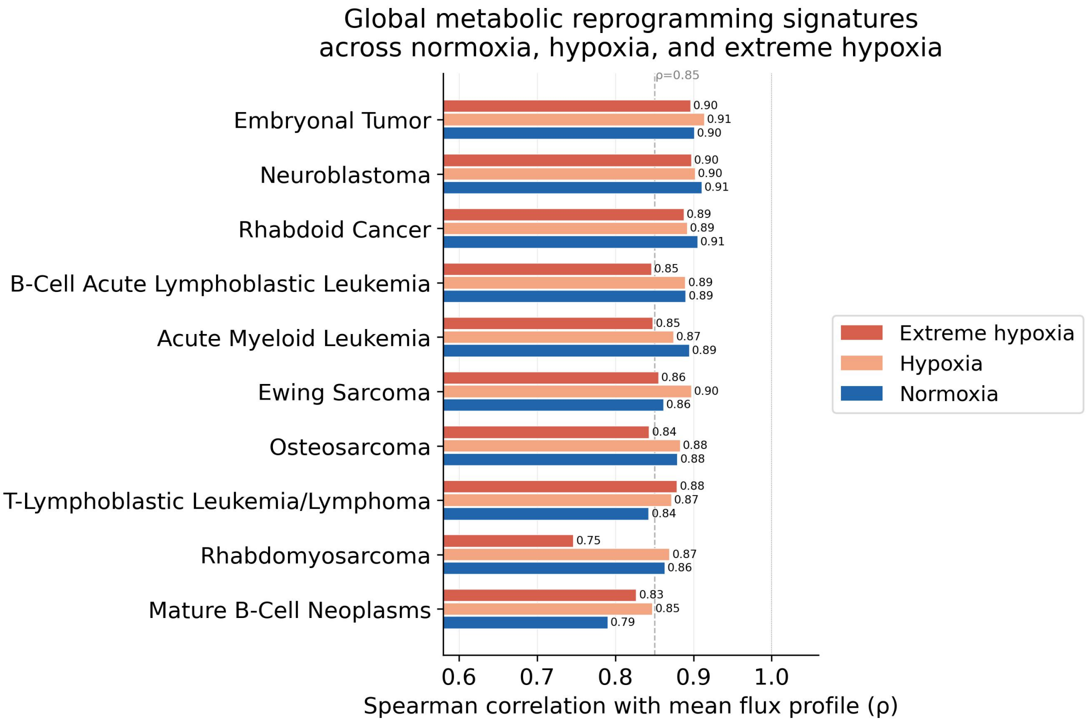

Figure S1. Consistency of metabolic reprogramming signatures in 10 pediatric cancers across normoxia, hypoxia, and extreme hypoxia. Spearman correlation coefficients (ρ) between each pediatric cancer's median normalized pathway flux fractions and the mean of all cancer categories, computed separately for normoxia (blue), hypoxia (orange), and extreme hypoxia (red). Cancer types are ranked by the mean ρ across all three oxygen classes. The dashed reference line indicates a chosen baseline value, ρ=0.85.

4 DATA AND FEATURES USED IN MACHINE LEARNING ALGORITHMS

We used 6 different machine learning algorithms, like Logistic Regression, Support Vector Machines, Random Forest (RF), GradientBoosting (GB), LightGBM(LGBM), and HistGradientBoosting algorithms (HGB). We created the features using pFBA data (flux active reactions) of 147 GEMs of 10 pediatric cancers for 10 oxygen gradients (1 to 0.001). The total number of samples is 1470. We have categorized the features into the following groups.

- The primary feature is the pathway contribution to the total flux. In the Recon 3D derived models, reactions are grouped into metabolic subsystems or pathways (Brunk et al., 2018). Ratio of fluxes of reactions in pathways to the total flux across each fraction in every model. This helps interpret the allocation of total flux to essential pathways - 89 flux features generated from this.
- Second group is the flux of compartment-wise and total reactive species demand reaction, normalized to total flux - 7 features generated from this.

  Compartment-wise demands were added as features to delineate the organellar reactive species burden. The RS demand reactions are shown for understanding. Let us say that hydrogen sulfide is present in two compartments in total - cytosol [c] and mitochondria [m], the compartmental demand reactions are shown as

$H_2S[c] \rightarrow H_2S[total]$
$H_2S[m] \rightarrow H_2S[total]$

Then, a demand reaction was added to denote the overall demand,
$H_2S[total] \rightarrow$
All the features generated up to this point were normalized by the total flux in each model at each fraction.

- Ratio of oncometabolite secretion flux normalized to total exchange flux - We created a panel of 10 well-known oncometabolites, and summed their fluxes to calculate the contribution of oncometabolite exchange activity compared to total exchange - 1 feature from this.
- We identified certain pathway trade-offs that denote the prevalence of major cancer hallmarks and calculated log-ratios (Schiliro and Firestein, 2021; Jiang et al., 2014; Santiappillai et al., 2025) - 3 features were generated from this list.

  1. Glutaminolysis/total TCA input to calculate glutaminolysis-driven anaplerosis
  2. Pentose phosphate pathway/glycolysis to calculate the fraction of glucose carbon that goes in PPP
  3. Features added to investigate one-carbon metabolism - folate from serine/total folate to inspect the serine dependence

Reactions and pathways associated with artificial reactions, such as exchanges and demands of metabolites, were removed as features, while sinks were retained. Oxidative phosphorylation pathway was removed as a feature because we added experimental oxygen consumption rate to the cytochrome oxidase reaction, and constrained it to simulate normoxic to extreme hypoxic states. All the features are scaled before proceeding to ML analysis.

5 EVALUATION OF TOP ML CLASSIFIERS

We have shown the performance evaluation of the top first, second, and third ML classifiers from the test set in Figures LGBM: S2, HGB: S3, and RF: S4. We have plotted a confusion matrix and an AUC-ROC curve to evaluate their performances in terms of classification of the three oxygen states. The macro-F1 and AUC scores of LGBM are greater than those of the other two algorithms. We also observe that hypoxia has a comparatively slightly lower AUC value in all 3 classifiers, and this is attributed to the fact that hypoxia is a middle gradient and thereby shares some traits with both normoxia and extreme hypoxia.

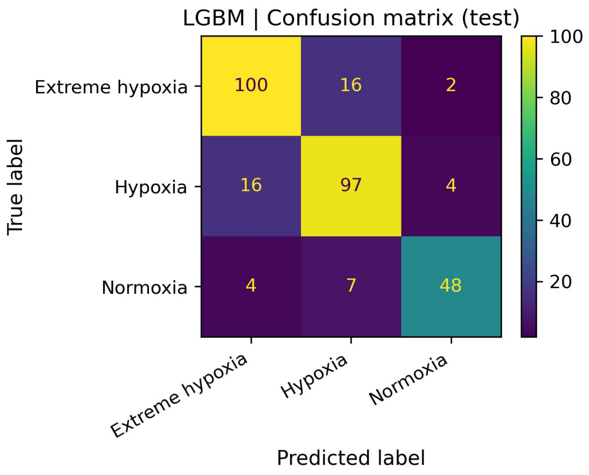

(A) Confusion matrix of LightGBM classification predictions. Confusion matrix for each oxygen state showing classification performance, where rows represent true labels and columns represent predicted labels. Correct classifications are along the diagonal, and misclassifications are off-diagonal.

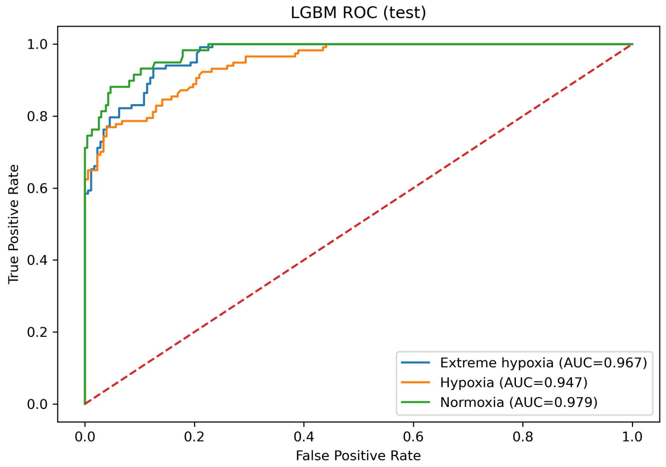

(B) Receiver Operating Characteristic (ROC) curves for normoxia, hypoxia, and extreme hypoxia with corresponding Area Under the Curve (AUC) values by LightGBM.

Figure S2. Performance evaluation of the LightGBM (LGBM) classifier on the test dataset. Confusion matrix and one-vs-rest ROC–AUC curves for LightGBM-based multi-class classification of oxygen states, evaluated using macro-averaged metrics.

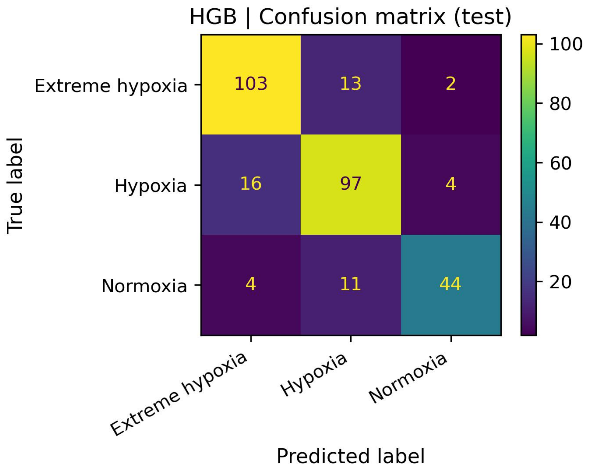

(A) Confusion matrix of HGB classification predictions. Confusion matrix for each oxygen state showing classification performance, where rows represent true labels and columns represent predicted labels. Correct classifications are along the diagonal, and misclassifications are off-diagonal.

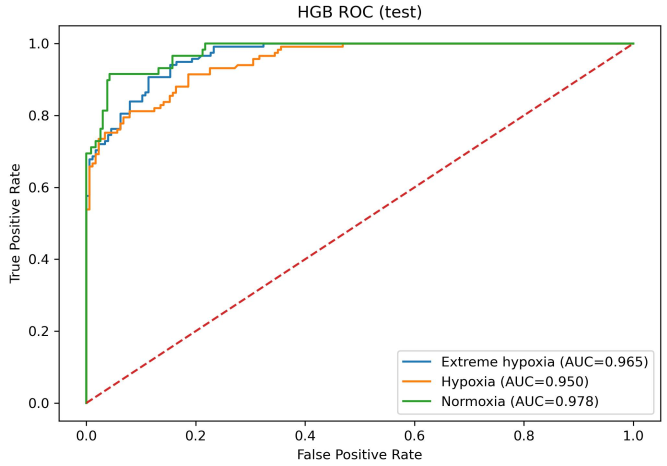

(B) Receiver Operating Characteristic (ROC) curves for normoxia, hypoxia, and extreme hypoxia with corresponding Area Under the Curve (AUC) values by HGB.

Figure S3. Performance evaluation of the HGB classifier on the test dataset. Confusion matrix and one-vs-rest ROC–AUC curves for HGB-based multi-class classification of oxygen states, evaluated using macro-averaged metrics.

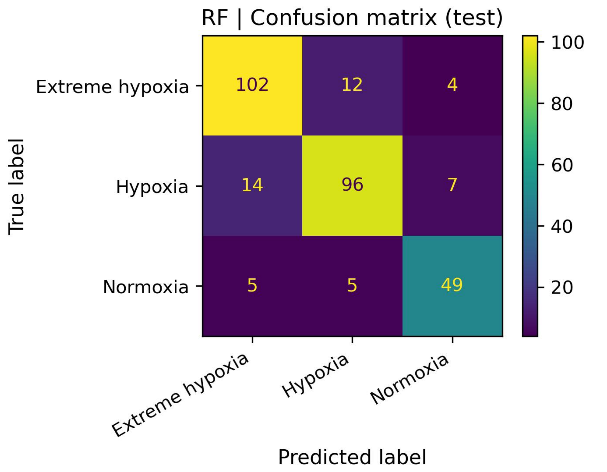

(A) Confusion matrix of RF classification predictions. Confusion matrix for each oxygen state showing classification performance, where rows represent true labels and columns represent predicted labels. Correct classifications are along the diagonal, and misclassifications are off-diagonal.

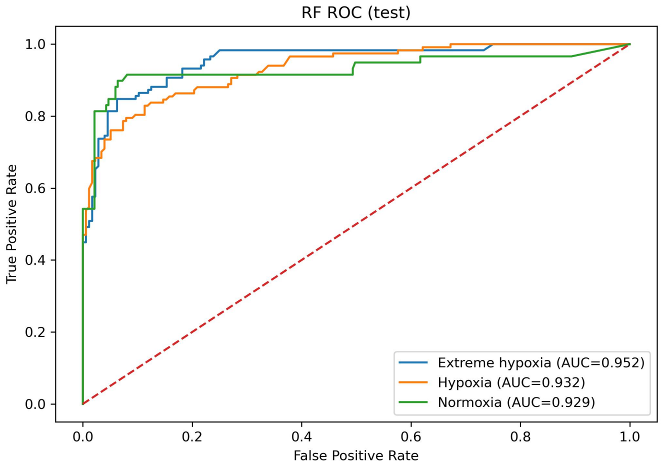

(B) Receiver Operating Characteristic (ROC) curves for normoxia, hypoxia, and extreme hypoxia with corresponding Area Under the Curve (AUC) values by RF.

Figure S4. Performance evaluation of the RF classifier on the test dataset. Confusion matrix and one-vs-rest ROC–AUC curves for RF-based multi-class classification of oxygen states, evaluated using macro-averaged metrics.

6 TRAJECTORY PLOTS OF PENTOSE PHOSPHATE PATHWAY (PPP) BRANCHING FROM GLYCOLYSIS

We observe that PPP branching reactions peak at the initial hypoxic fraction, before decreasing towards extreme hypoxia (Figure S5A). Glycolysis remains steady from normoxia till hypoxia, and reduces under extreme hypoxia (Figure S5B). Negative logarithmic values indicate that the denominator is always high; glycolysis reaction fluxes do not become less than PPP branching. Only under extreme hypoxia, we see some preference to PPP compared to the earlier states (Figure S5C).

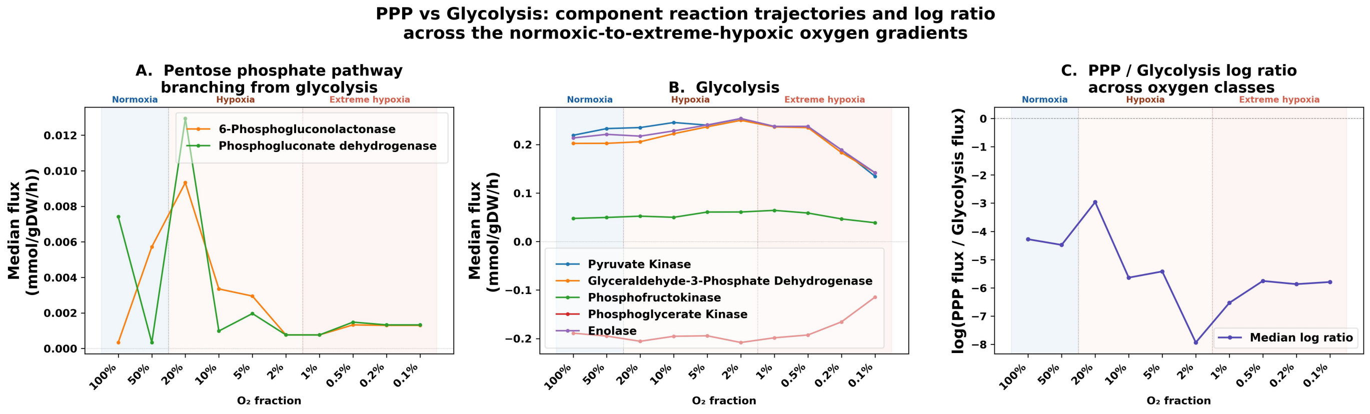

Figure S5. Trajectory of reactions involved in PPP branching from glycolysis, glycolysis reactions, and ratio of PPP branching from glycolysis in terms of logarithmic ratios

REFERENCES

Bernauer, C., Man, Y. S., Chisholm, J. C., Lepicard, E. Y., Robinson, S. P., and Shipley, J. M. (2021). Hypoxia and its therapeutic possibilities in paediatric cancers. *British journal of cancer* 124, 539–551

Bhalla, K., Jaber, S., Nahid M, N., Underwood, K., Beheshti, A., Landon, A., et al. (2018). Role of hypoxia in diffuse large b-cell lymphoma: Metabolic repression and selective translation of hk2 facilitates development of dlbcl. *Scientific reports* 8, 744

Brunk, E., Sahoo, S., Zielinski, D. C., Altunkaya, A., Dräger, A., Mih, N., et al. (2018). Recon3d enables a three-dimensional view of gene variation in human metabolism. *Nature biotechnology* 36, 272–281

Jiang, P., Du, W., and Wu, M. (2014). Regulation of the pentose phosphate pathway in cancer. *Protein & cell* 5, 592–602

Kumar S, P., Sridhar, S., Alsmadi, N., Mahadevan, R., and Bhatt, N. (2026). Generalist method to reconstruct metabolic networks from multi-omics data at large-scale. *bioRxiv* doi:10.64898/2026.04.02.716249

Santiappillai, N. T., Cao, Y., Hakeem-Sanni, M. F., Yang, J., Quek, L.-E., and Hoy, A. J. (2025). Pathway metabolite ratios reveal distinctive glutamine metabolism in a subset of proliferating cells. *Molecular Systems Biology* , 1–21

Schiliro, C. and Firestein, B. L. (2021). Mechanisms of metabolic reprogramming in cancer cells supporting enhanced growth and proliferation. *Cells* 10, 1056

Széliová, D., Ruckerbauer, D. E., Galleguillos, S. N., Petersen, L. B., Natter, K., Hanscho, M., et al. (2020). What CHO is made of: Variations in the biomass composition of chinese hamster ovary cell lines. *Metabolic Engineering* 61, 288–300
